# Toward improving retention in HIV care after pregnancy: lessons from a post-pandemic cohort in the United States

**DOI:** 10.3389/fmed.2025.1561490

**Published:** 2025-06-27

**Authors:** Alexandra H. Latham, Andrea Lugo Morales, Elizabeth Barba Gutierrez, Michael Jochum, Sarah Conrad, Eva H. Clark, Kassandra Goytia, Megan Alam, Jessica Gerard, Jennifer McKinney

**Affiliations:** ^1^Department of Obstetrics and Gynecology, Baylor College of Medicine, Houston, TX, United States; ^2^Department of Obstetrics and Gynecology, Harris Health System, Houston, TX, United States; ^3^Department of Obstetrics and Gynecology, Texas Children's Hospital, Houston, TX, United States; ^4^Department of Medicine (Infectious Diseases) and Pediatrics (Tropical Medicine), Baylor College of Medicine, Houston, TX, United States

**Keywords:** HIV, pregnancy, postpartum, retention, barriers

## Abstract

**Introduction:**

People living with HIV (PLWH) often experience low rates of retention in HIV care (RIC) and suboptimal viral suppression postpartum. Understanding contemporary barriers to RIC is crucial to identify risk factors for loss to care and thereby improve support during this vulnerable transition. This work aimed to identify factors associated with adequate RIC, defined as two HIV care visits ≥90 days apart in the first year postpartum.

**Methods:**

Electronic records were retrospectively reviewed for PLWH who delivered from 2019 to 2023 and received prenatal care within a single county health system. Variables were collected related to both maternal and neonatal HIV and obstetric or pediatric care. Variables were analyzed using descriptive statistics, and Kaplan-Meier curves were used to assess viral suppression during pregnancy and the first year postpartum. A Random Forest machine learning model was used to determine variables of relative importance for prediction of adequate RIC. Multivariable logistic regression was used to evaluate impact of identified variables on RIC.

**Results:**

Of 182 pregnancies, sixty individuals (33%) achieved adequate postpartum RIC. Adequate RIC correlated with year of delivery (*p* = 0.018), attending at least two obstetrical postpartum visits (*p* = 0.025), viral suppression at initial prenatal visit (*p* = 0.030), and shorter duration between pregnancy visit and HIV care visits before and after pregnancy (*p* < 0.001). Viral suppression was generally excellent at time of delivery (99.4%). However, viral loads rebounded after delivery, with 66.8% suppressed at 12 months postpartum. Random Forest modeling identified several clinical and social factors with relative importance for prediction of RIC. Multivariable logistic regression supported above findings with significant decreased odds of adequate RIC based on year of delivery [2021 aOR 0.306 (0.097–0.956), 2022 0.146 (0.046–0.458), 2023 0.071 (0.011–0.455)], higher viral load at initial prenatal visit [aOR 0.038 (0.002–0.889)], and longer duration between last HIV care visit and first pregnancy visit [aOR 0.419 (0.176–0.998)].

**Discussion:**

Postpartum RIC was suboptimal in this contemporary US single-site cohort. Engagement in prenatal and postpartum obstetric care predicted improved postpartum RIC. Further qualitative research is essential to improve deeper understanding of patterns of engagement perinatally in order to develop effective interventions to improve support for individuals during this difficult transition.

## 1 Introduction

Consistent and routine engagement in HIV healthcare, referred to as retention in care (RIC), is crucial for the wellbeing of people living with HIV (PLWH) (1, 2). Health Resources and Services Administration (HRSA) and Institute of Medicine (IOM) define RIC as two or more medical visits separated by >90 days within 1 year, while the U.S. Centers for Disease Control and Prevention (CDC) defines RIC as two or more CD4 cell counts or HIV viral load tests performed at least three months apart (3–5). While the National HIV/AIDS Strategy goal is 90% RIC, the CDC estimated RIC for U.S. PLWH to be only 50–58% from 2010–2022 (3). PLWH retained in care have better viral suppression and health outcomes compared to those with suboptimal RIC, who tend to have lower CD4 counts and a higher incidence of AIDS-defining events (6).

RIC, and closely correlated viral suppression, is particularly important for pregnant and postpartum PLWH to prevent perinatal HIV transmission. However, the transition between pregnancy care (a period characterized by high engagement and excellent viral suppression) (7) and postpartum care is a vulnerable time for many PLWH. Parents have numerous competing priorities and face myriad challenges to RIC. Previously identified barriers to RIC include lack of social support (in part due to HIV-related stigma), mental health challenges, transportation availability, and childcare and work responsibilities (8–10). Regional differences in public health policies and infrastructure, in particular variable healthcare coverage through Medicaid and safety net programs, also impact RIC (11). Given these challenges, it is unsurprising that RIC rates vary widely (39–76% between 2015–2019) in the first 12 months postpartum (7, 11–15). A previous study from 2014 of a similar regional population found that the loss to HIV primary care providers (PCP) was 39% in the first year postpartum with only 36% attending a minimum of one clinical visit to PCP every 6 months within the first year after delivery (16). Suboptimal RIC during the postpartum period is associated with lower antiretroviral therapy (ART) adherence, viral rebound, poorer health outcomes, and higher chance of entering the next pregnancy without viral suppression (17, 18). Further, viral suppression and engagement in care is particularly important for postpartum PLWH who choose to breast/chestfeed their infants as it minimizes the risk of lactational HIV transmission to the infant (19).

Given these negative consequences of poor RIC on maternal and neonatal outcomes, it is important to identify recent trends in and barriers to RIC among pregnant and postpartum PLWH. This study aimed to describe the contemporary factors contributing to inadequate postpartum RIC within a US county healthcare system.

## 2 Materials and methods

This retrospective cohort study reviewed all pregnant PLWH who delivered between January 1, 2019, and February 28, 2023, within Harris Health, a large county healthcare system that includes the Houston, TX metropolitan area.

We included pregnant PLWH who were diagnosed with HIV either before or during the reviewed pregnancy, delivered a liveborn neonate or had a loss after 14 weeks gestation during the study time-period, received prenatal care within Harris Health via a Baylor College of Medicine (BCM) physician, and planned to obtain HIV care within Harris Health after delivery. We excluded pregnant PLWH with pregnancy loss prior to 14 weeks gestation or with a repeat pregnancy within 12 months postpartum as well as anyone who was incarcerated during pregnancy or within 12 months postpartum, transferred out of Harris Health or were lost to follow-up before delivery, or intended to receive postpartum HIV care outside of Harris Health.

We reviewed the electronic medical record and collected the variables from the following categories: demographics, obstetrical care (pregnancy, delivery, and postpartum factors), HIV-related care (HIV care visits, laboratory data, antiretroviral therapy), and neonatal care (neonatal ICU admission, testing results, pediatric retrovirology surveillance visits). We evaluated adequacy of prenatal care using the Adequacy of Prenatal Care Utilization (APNCU) Index (20) and approximated social vulnerability using the CDC and Agency for Toxic Substances and Disease Registry Social Vulnerability Index (CDC/ATSDR SVI) (21). Neonatal and postpartum data was collected for at least 12 months after delivery. Data was de-identified, coded, and entered data into a secure electronic REDCap database.

We defined adequate postpartum RIC as attendance at two or more HIV care visits at least 90 days apart within the first 12 months postpartum consistent with HRSA and IOM definitions of RIC (5). Labs are routinely performed at all HIV care visits, but laboratory data alone was not used to define outcome of interest in this study. Viral suppression was defined as HIV-1 RNA (viral load) < 50 copies/mL.

Categorical variables were described as numbers with percentages, and chi-square tests or Fisher's exact tests were used for testing significance. Continuous data were described as means with standard deviation (SD) if normally distributed, or medians with interquartile ranges (IQRs) when data were skewed. Unpaired *t*-tests and Mann-Whitney U tests were used for testing significance for continuous data. Kaplan-Meier curves and inverse Kaplan-Meier curves were used to assess pregnancy and postpartum viral suppression. No imputation was made for missing data.

A Random Forest machine learning model was used to assess the ability of each variable to predict adequate RIC. Before model training, data was preprocessed to handle missing values, outliers, and perform necessary transformations (such as normalization or scaling) to ensure that all variables were in a suitable format for machine learning algorithms. Train control objects were set up for 10- fold 10 repeat k-fold cross-validation, helping in evaluating the model's performance and selecting the best hyperparameters. Following preprocessing, data was split into two sets: 80% for training and 20% for testing. We used the training set for model optimization to determine ability to predict RIC and reserved the testing set for evaluation of the final model's performance. The model was tuned to optimize the ROC (Receiver Operating Characteristic) metric. After training, the model's performance was visualized and variable importance was determined. The machine learning model was then trained on the variables of interest and employed to determine the relative importance of each variable on predicting adequate RIC.

To assess the association between the variables identified by the Random Forest model with the highest relative importance, we fit a multivariable logistic regression model. Following model fitting, the estimated coefficients were exponentiated to obtain adjusted odds ratios (aOR) along with 95% confidence intervals (CI) for each fixed effect.

All analyses were performed using R Studio software (Integrated Development for R. R Studio, PBC, Boston, MA).

The BCM institutional review board (IRB) approved this study and waived informed consent (H-55046).

## 3 Results

**Figure 1:**
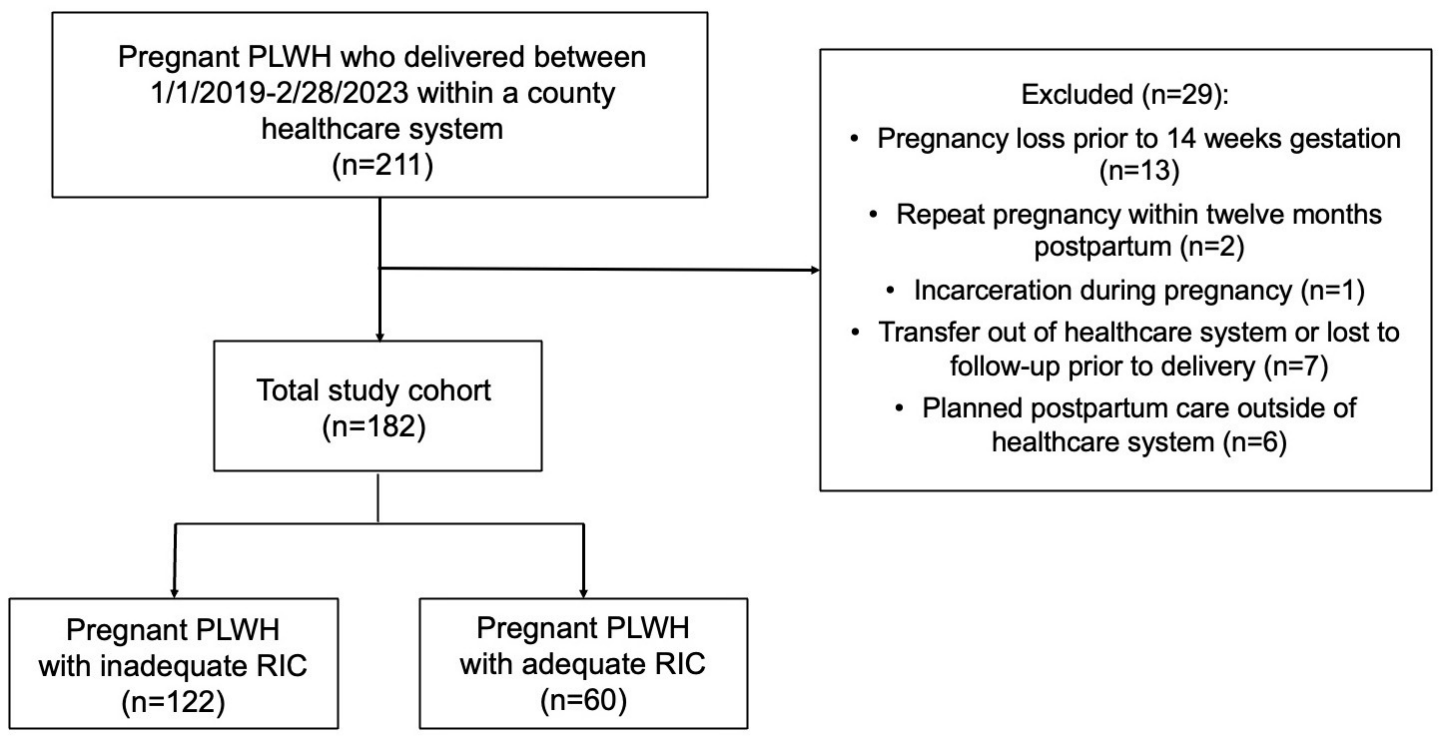
Cohort diagram. A total cohort of 182 pregnant people living with HIV (PLWH) met study criteria. Of these, only 33% had adequate postpartum retention in care (RIC), defined as 2 visits ≥90 days apart in the first 12 months post-delivery.

A total of 211 pregnant PLWH delivered between January 1, 2019 and February 28, 2023 within the specific county healthcare system. Of these, 29 were excluded, leaving 182 pregnant PLWH who met study criteria. Only 33% (60) PLWH were retained in care at 12 months postpartum (Figure 1).

**Figure 2:**
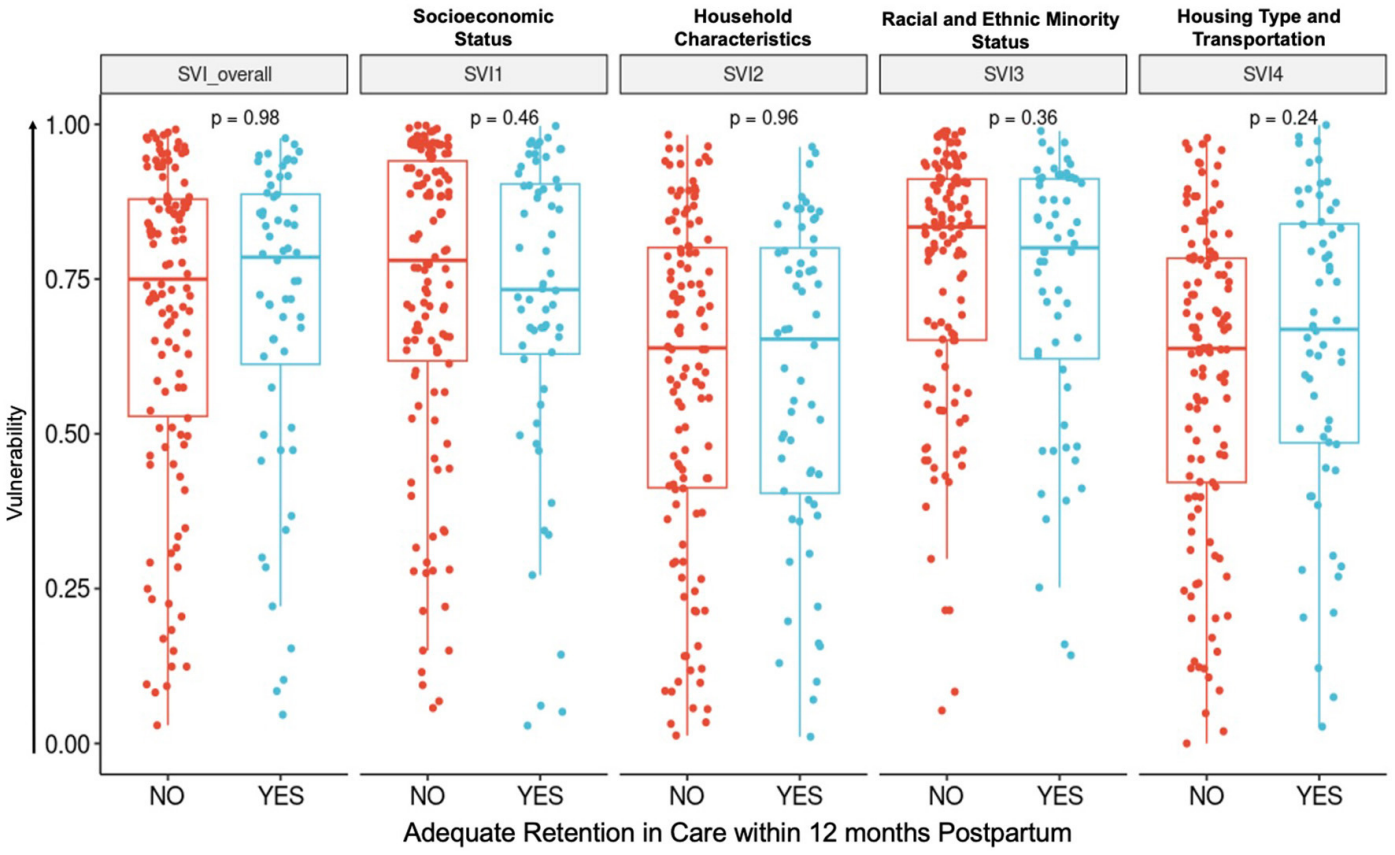
Social vulnerability. Centers for Disease Control and Prevention (CDC) Social Vulnerability Index (SVI) demonstrates high vulnerability throughout the entire cohort. Vulnerability is ranked in comparison to vulnerability across all other census tracts in the state.

PLWH with and without adequate postpartum RIC had similar demographic and pregnancy characteristics (Table 1). The mean age was 30 years (range 16–45 years), and the majority were multiparous (137, 75%). Most were born in the United States (followed by Africa and then Latin America), spoke English as their preferred language, had government-funded public insurance, were unemployed, and had at least a high school equivalent level of education. Substance use (31, 17%) and mental health diagnoses (78, 42%) occurred at similar frequencies in both groups. The SVI was high in both RIC groups—with greater vulnerability than 75% of other census tracts in the state for those with adequate RIC and greater vulnerability than 78% of other census tracts for those with inadequate RIC (Figure 2).

**Table 1:** Demographic and pregnancy characteristics of the cohort by adequacy of postpartum retention in HIV care (RIC).

**Characteristic**	**Total study population *N* = 182**	**Inadequate RIC *N* = 122**	**Adequate RIC *N* = 60**	***p*-value**
Age at Delivery^*^	30.3 (6.0)	30.6 (6.1)	29.8 (5.8)	0.369
Age < 20 years	9 (4.9)	7 (5.7)	2 (3.3)	0.734
Multiparous	137 (75.3)	95 (77.9)	42 (70.0)	0.330
Living children^**^	1.5 (2.0)	1.9 (1.6)	1.5 (1.3)	0.553
Current substance use	31 (17.0)	24 (19.7)	7 (11.7)	0.254
Mental health condition	78 (42.9)	55 (40.1)	23 (38.3)	0.481
Pre-pregnancy BMI^*^	22 (14.4)	21.7 (15.2)	22.9 (12.6)	0.561
**Region of birth**				0.270
United States	106 (58.2)	76 (62.3)	30 (50.0)	
Africa	39 (21.4)	26 (21.3)	13 (21.7)	
Latin America	31 (17.0)	17 (13.9)	14 (23.3)	
**Ethnicity and race**				0.132
Hispanic	47 (25.8)	30 (24.6)	17 (28.3)	
Non-Hispanic White	7 (3.8)	5 (4.1)	2 (3.3)	
Non-Hispanic Black	121 (66.5)	84 (68.9)	37 (61.7)	
**Primary language**				0.958
English	137 (75.3)	95 (77.9)	42 (70.0)	
Spanish	28 (15.4)	14 (11.5)	14 (23.3)	
**Insurance**				0.450
Medicaid/medicare	126 (69.2)	89 (73.0)	37 (61.7)	
Childrens health insurance program	28 (15.4)	16 (13.1)	12 (20.0)	
Private insurance	19 (10.4)	12 (9.8)	7 (11.7)	
Self-pay	9 (4.9)	5 (4.1)	4 (6.7)	
**Education level**				0.679
Less than high school equivalent	38 (20.9)	26 (21.3)	12 (21.7)	
Equal to or more than high school equivalent	106 (58.3)	69 (56.6)	37 (61.7)	
Employment Status, Employed	63 (34.6)	42 (34.4)	21 (35.0)	0.561
Marital Status, Single	104 (57.1)	74 (60.6)	30 (50.0)	0.198
**Social vulnerability (CDC/ATSDR SVI)** ^**^				
SVI 1: Socioeconomic status	0.77 (0.3)	0.78 (0.3)	0.73 (0.3)	0.458
SVI 2: Household characteristics	0.64 (0.4)	0.64 (0.4)	0.65 (0.4)	0.959
SVI 3: Racial and ethnic minority status	0.82 (0.3)	0.83 (0.3)	0.8 (0.3)	0.364
SVI 4: Housing and transportation	0.65 (0.4)	0.64 (0.4)	0.67 (0.4)	0.237
Overall	0.77 (0.3)	0.75 (0.4)	0.79 (0.4)	0.983
Adequate prenatal care (APNCU, Observed: Expected ≥ 80%)	56 (30.8)	35 (28.7)	21 (35.0)	0.486
Gestational age at initial prenatal visit (day)^**^	90 (62.0)	91 (66.0)	81 (50.0)	0.223
Number of Prenatal Visits^*^	8.9 (3.3)	8.8 (3.3)	9.2 (3.3)	0.372
Participated in group prenatal care	73 (40.1)	50 (41.0)	23 (40.4)	0.915
Term delivery	152 (83.5)	101 (82.8)	51 (85.0)	0.868
Mode of delivery, vaginal	95 (52.2)	59 (49.4)	36 (60.0)	0.282
Cesarean delivery indication: HIV	4 (2.2)	4 (3.3)	0 (0.0)	0.379
Postpartum contraception use	143 (78.6)	93 (76.2)	50 (83.3)	0.412
NICU Admission	63 (34.6)	48 (40.3)	15 (25.0)	0.091
**Year of delivery**				**0.018**
**2019**	**39 (21.4)**	**20 (16.4)**	**19 (31.7)**	
**2020**	**32 (17.6)**	**17 (13.9)**	**15 (25.0)**	
**2021**	**35 (19.2)**	**24 (19.7)**	**11 (18.3)**	
**2022**	**57 (31.3)**	**45 (36.9)**	**12 (20.0)**	
**2023**	**17 (9.3)**	**14 (82.4)**	**3 (17.6)**	

Data is represented as total number (percentage) unless otherwise specified: ^*^mean (SD); ^**^median (interquartile range). Significant associations are bolded. Display of select categories and intermittent item missingness may result in columns summing less than expected. APNCU, Adequacy of Prenatal Care Utilization index; BMI, body mass index; HIV, human immunodeficiency virus; NICU, neonatal intensive care unit; RIC, postpartum retention in HIV care; SVI, Centers for Disease Control and Prevention and Agency for Toxic Substances and Disease Registry Social Vulnerability Index.

**Figure 3:**
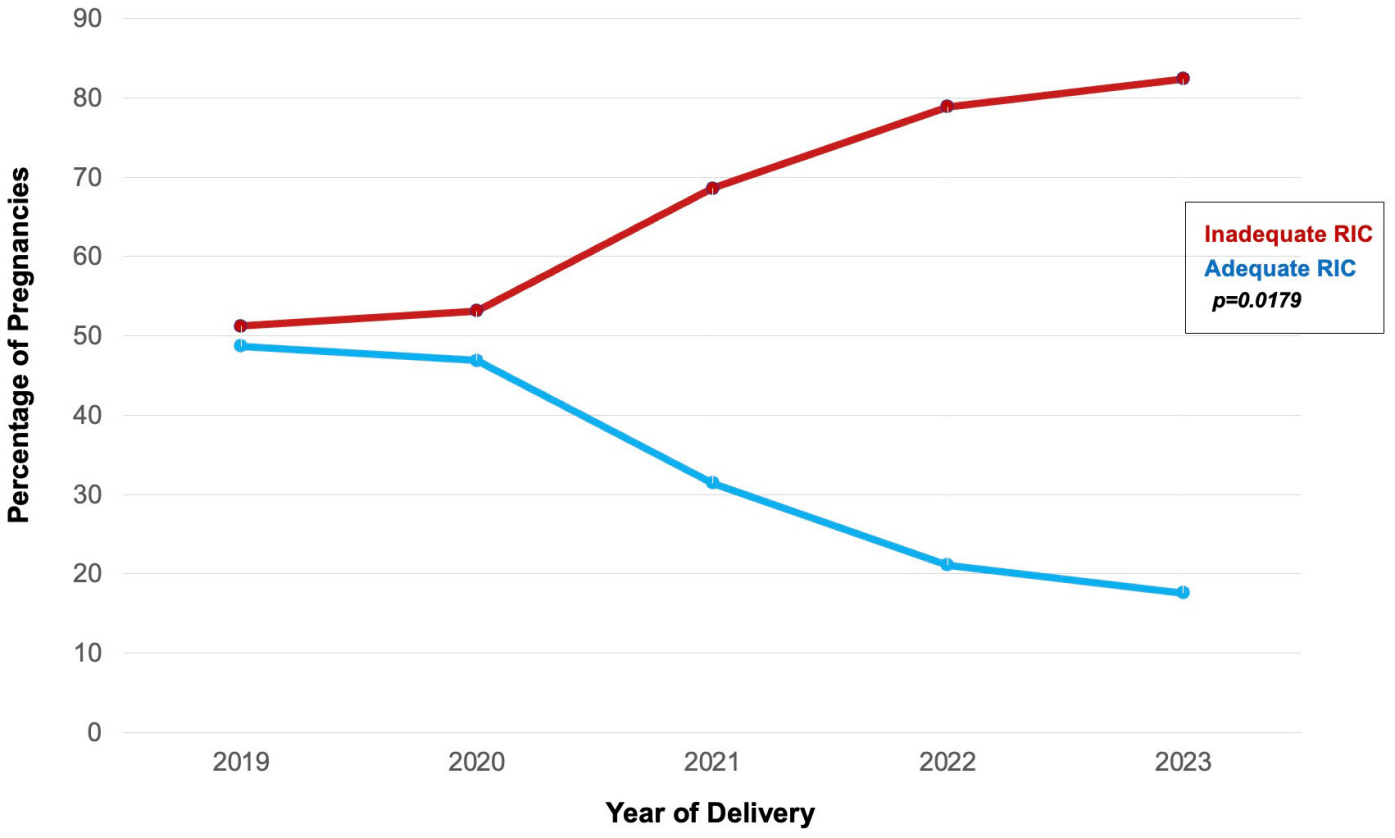
Retention in care by year of delivery. Post-pandemic years showed a significant increase in inadequate postpartum retention in care (RIC).

Pregnancy-related hypertension, anemia, and preterm delivery were the most common pregnancy complications. Most entered prenatal care in the first trimester (12–13 weeks), but only 56 (30%) had adequate prenatal care as measured by Adequacy of Prenatal Care Utilization (APNCU) Index. Four cesarean deliveries were performed due to viral load >1,000 copies/mL, all of whom subsequently had inadequate postpartum RIC. Importantly, we found a significant relationship between adequate RIC and year of delivery; those who delivered after 2020 were much more likely to have inadequate postpartum RIC, with the disparity increasing each year (*p* = 0.018) (Table 1; Figure 3).

**Figure 4:**
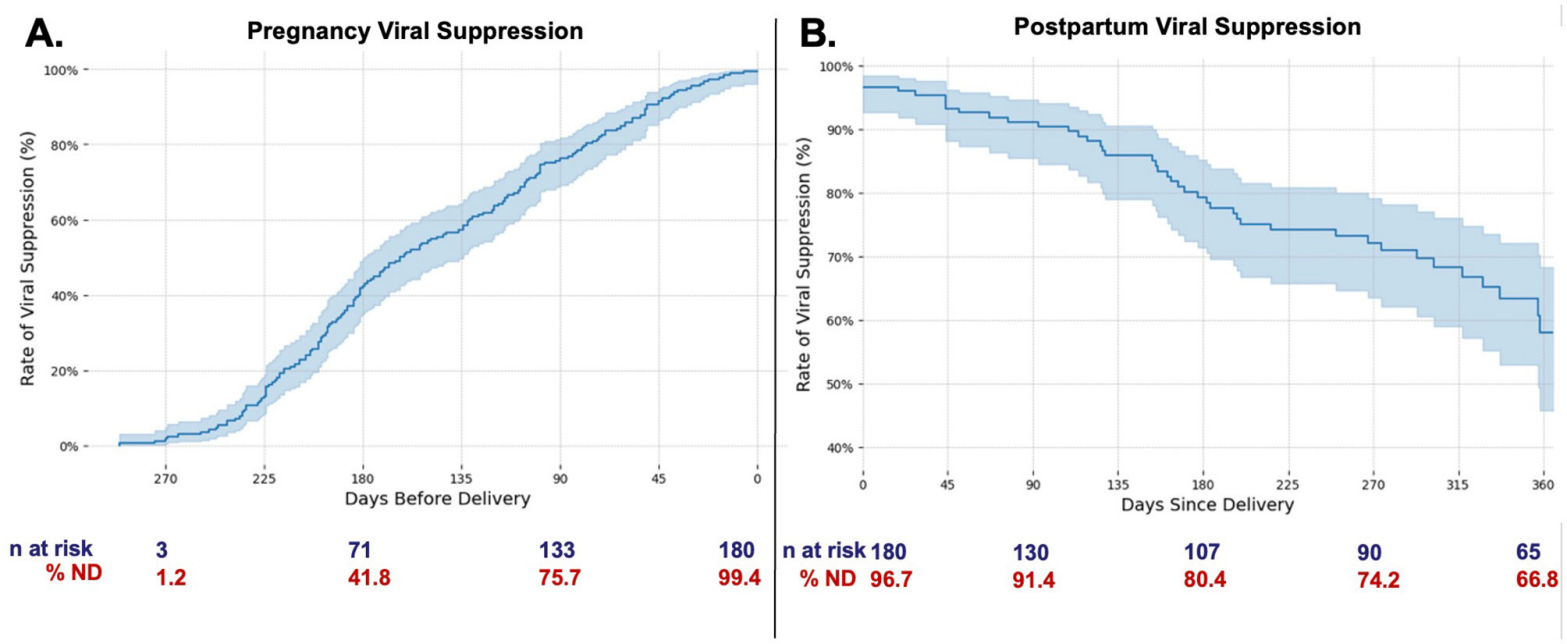
Viral suppression during pregnancy and postpartum. Viral suppression (having an undetectable viral load, <50 copies/mL) increases during pregnancy **(A)**, then decreases steadily after delivery **(B)**. Time 0 indicates date of delivery. Note the drop off in lab values available following delivery, consistent with inadequate retention in care. ND, viral load not detectable; <50 copies/mL.

Most pregnant PLWH were diagnosed with HIV prior to the index pregnancy (152, 83%), nine (4%) of whom had acquired HIV perinatally. The majority (142, 78%) had been prescribed ART prior to pregnancy, including 138 (76%) with a single tablet regimen, most commonly bictegravir/emtricitabine/tenofovir alafenamide (73, 40%). Signs of engagement in pre-pregnancy HIV care (viral suppression at entry to prenatal care) and postpartum obstetric care (attendance at postpartum visits) were significantly associated with adequate postpartum RIC (*p* = 0.030 and *p* = 0.025, respectively). A shorter time interval between delivery and first postpartum HIV care visit was also associated with adequate postpartum RIC (222 days for those with inadequate RIC vs. 115 for adequate RIC, *p* < 0.001) (Table 2). Viral suppression improved greatly over the course of pregnancy as 180 (99.4%) PLWH were optimally suppressed (viral load < 50 copies/mL) at delivery. However, viral suppression decreased steadily after delivery with only 122 (67%) PLWH still virally suppressed at 12 months postpartum (Figure 4).

**Table 2:** Maternal characteristics and immunological and virological characteristics by adequacy of postpartum retention in HIV care (RIC).

**Characteristic**	**Total study population *N* = 182**	**Inadequate RIC *N* = 122**	**Adequate RIC *N* = 60**	***p*-value**
Age at HIV diagnosis^*^	23.6 (8.1)	23.9 (7.9)	23 (8.7)	0.494
Perinatal HIV diagnosis	9 (4.9)	4 (3.3)	5 (8.3)	0.265
HIV diagnosis before pregnancy	152 (83.5)	102 (86.6)	50 (83.3)	1.000
Days from last HIV care visit to initial prenatal visit^**^	158 (236.0)	178 (305.0)	146 (170.0)	0.203
**Viral load (HIV-1 RNA)**				
Last pre-pregnancy viral load < 50 copies/mL	24 (13.2)	16 (13.1)	8 (13.3)	1.000
**Initial prenatal visit viral load** ** < 50 copies/mL**	**99 (54.4)**	**59 (48.4)**	**40 (66.7)**	**0.030**
Delivery viral load < 50 copies/mL	152 (83.5)	98 (80.3)	54 (90.0)	0.150
**CD4 Count**				
CD4 at initial prenatal visit^*^	575 (305.0)	560 (310.0)	606 (294.0)	0.334
CD4 < 200 at initial prenatal visit	22 (12.1)	17 (13.9)	5 (8.3)	0.418
Antiretroviral therapy Initiation before index pregnancy	142 (78.0)	93 (76.2)	49 (81.7)	0.521
ART single pill regimen use	138 (75.8)	92 (75.4)	46 (76.7)	0.998
Bictegravir-containing	73 (40.1)	54 (44.3)	19 (31.7)	
**Attended** **>2 postpartum obstetric visits**	**124 (68.1)**	**76 (62.3)**	**48 (80.0)**	**0.025**
**Last postpartum visit within 12m viral load** ** < 50 copies/mL**	**113 (62.1)**	**67 (54.9)**	**46 (76.7)**	**0.007**
**Days from delivery to first HIV care visit** ^**^	**130 (101.0)**	**222 (177.0)**	**115 (47.0)**	**< 0.001**
**Days between delivery and first HIV care visit**				**< 0.001**
**First HIV care visit** ** < 90 days from delivery**	**34 (18.7)**	**16 (13.0)**	**18 (30.0)**	
**First HIV care visit 90–180 days from delivery**	**57 (31.3)**	**20 (16.4)**	**37 (61.7)**	
**First HIV care visit** **>180 days from delivery**	**36 (19.8)**	**31 (25.4)**	**5 (8.3)**	
**Neonatal information**				
Confirmed neonatal HIV acquisition	1 (0.5)	1 (0.8)	0 (0.0)	Not calculated
**Neonatal retroviral surveillance visit attendance**				
Attended 2 week	128 (70.3)	84 (68.9)	44 (73.3)	0.653
Attended 2 month	108 (59.3)	66 (54.1)	42 (70.0)	0.058
Attended 4 month	95 (52.2)	58 (47.5)	37 (61.7)	0.102
Attended 18 month	50 (27.5)	28 (23.0)	22 (36.7)	0.076

Data is represented as total number (percentage) unless otherwise specified: ^*^mean (SD); ^**^median (interquartile range). Significant associations are bolded. Display of select categories and intermittent item missingness may result in columns summing less than expected. ART, antiretroviral therapy; HIV, human immunodeficiency virus; RIC, postpartum retention in HIV care.

Although not statistically significant, PLWH who did not have adequate postpartum RIC themselves were less likely to attend neonatal surveillance visits, particularly after the initial 2-week visit (Table 2). One neonate was confirmed to have acquired HIV perinatally during the study period; all other neonates tested negative.

**Figure 5:**
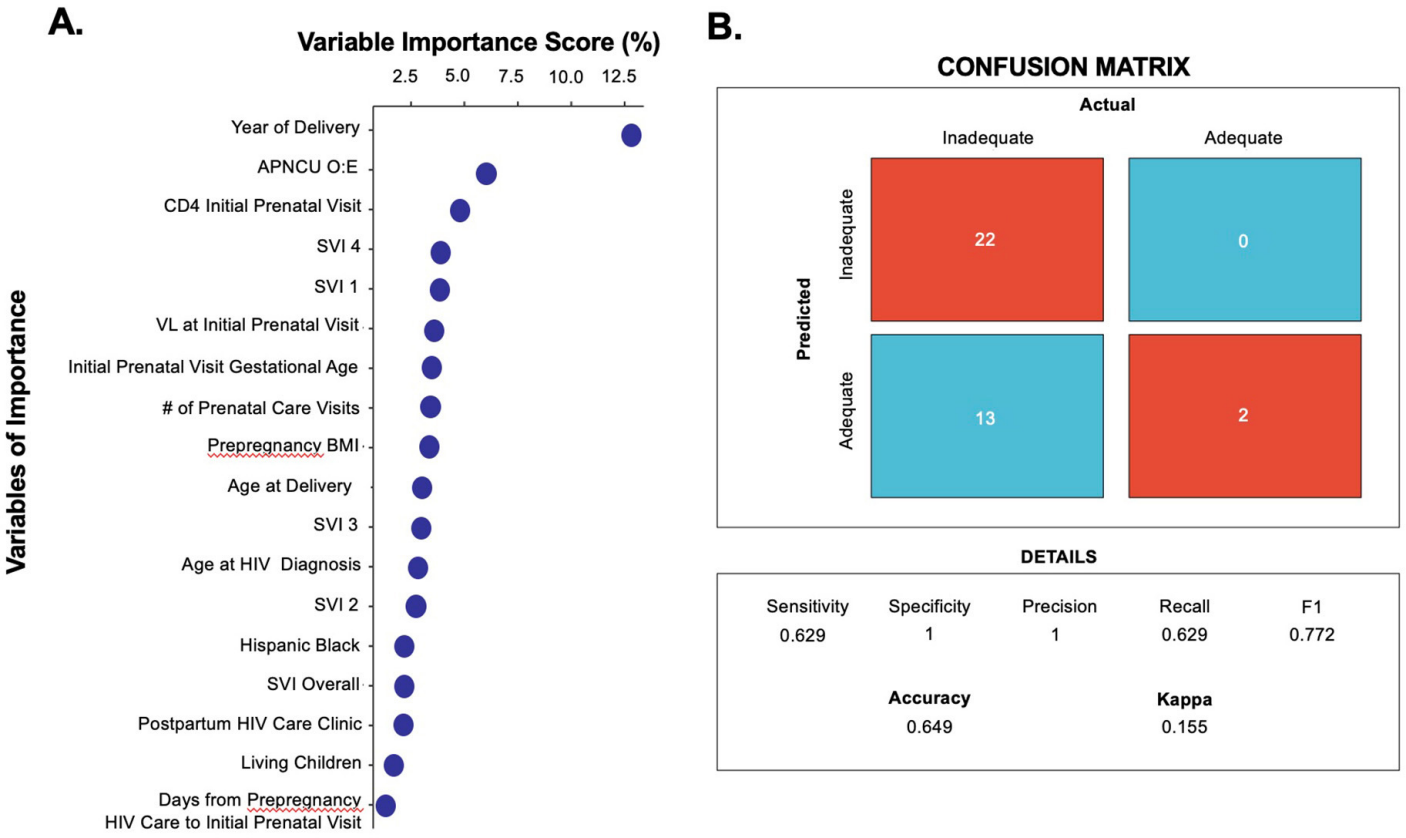
Random forest machine learning model. **(A)** Machine learning identifies variables of importance associated with adequate postpartum retention in care. **(B)** Confusion matrix of the model shows performance of the model in prediction of adequate retention in care. APNCU, Adequacy of Prenatal Care Utilization index; SVI, CDC Social Vulnerability Index; VL, viral load; BMI, body mass index.

The Random Forest training model had an accuracy of 64.9% with a sensitivity of 62.9% and specificity of 100% for prediction of postpartum RIC. Variables with the highest importance scores (i.e., those that contributed most to predicting adequacy of postpartum RIC) were year of delivery, viral load and CD4 count at initial prenatal care visit, Social Vulnerability Index (SVI) themes 1 (socioeconomic status) and 4 (housing and transportation), gestational age at initial prenatal care visit, number of prenatal visits, age at delivery and age at diagnosis, and adequate prenatal care as measured by APNCU index (Figure 5).

Multivariable logistic regression models supported similar themes: year of delivery was associated with a progressive 70–90% decreased chance of having adequate RIC, consistent with Figure 4. Measures that indicate challenges in engagement in care prior to delivery were associated with decreased likelihood of adequate RIC; higher viral load at initial prenatal visit [aOR 0.038 (0.002–0.889)], and longer duration between last HIV care visit and first pregnancy visit [aOR 0.419 (0.176–0.998)] (Table 3).

**Table 3:** Factors associated with adequate postpartum RIC in multivariable logistic regression modeling.

**Variable**	**aOR**	**95% CI**	***p*-value**
**Delivery year (reference year 2019)**			
2020	0.449	(0.139–1.451)	0.181
**2021**	**0.306**	**(0.097–0.965)**	**0.043**
**2022**	**0.146**	**(0.046–0.458)**	**0.001**
**2023**	**0.071**	**(0.011–0.455)**	**0.005**
Adequate prenatal care (APNCU, Observed: Expected ≥ 80%)	1.266	(0.480–3.340)	0.633
CD4 at initial prenatal visit	0.907	(0.578–1.424)	0.672
SVI Theme 4	0.524	(0.081–3.381)	0.497
SVI Theme 1	0.140	(0.010–1.975)	0.145
**Viral load (HIV-1 RNA) at initial prenatal visit**	**0.038**	**(0.002–0.889)**	**0.042**
Gestational age at initial prenatal visit	1.067	(0.646–1.762)	0.800
Number of prenatal visits	1.025	(0.422–2.490)	0.957
Pre-pregnancy BMI	1.009	(0.630–1.617)	0.970
Age at delivery	0.658	(0.344–1.257)	0.205
SVI theme 3	0.553	(0.251–1.218)	0.141
Age at HIV diagnosis	0.891	(0.477–1.665)	0.718
SVI Theme 2	0.376	(0.075–1.873)	0.232
Hispanic Black Race and ethnicity	0.000	*non-calc*.	*non-calc*.
SVI overall	34.948	0.1650–7410)	0.193
Living children	0.884	(0.559–1.397)	0.597
**Days from Last HIV care visit to initial prenatal visit**	**0.419**	**(0.176–0.998)**	**0.049**

Confidence intervals that do not overlap the null value of aOR = 1.0 are shown in bold. Multivariable logistic regression model included all of the demographic and clinical variables listed in the table. aOR, adjusted odds ratio; APNCU, Adequacy of Prenatal Care Utilization index; BMI, body mass index; CI, confidence interval; HIV, human immunodeficiency virus; non-calc, unable to be calculated; CI includes infinity; RIC, postpartum retention in HIV care; SVI, Centers for Disease Control and Prevention and Agency for Toxic Substances and Disease Registry Social Vulnerability Index.

## 4 Discussion

In this study examining the transition from obstetric to postpartum HIV care in a contemporary post-pandemic cohort of recently pregnant PLWH, we found that two-thirds had inadequate postpartum RIC. Identification of risk factors predicting postpartum RIC is critical to target interventions for individuals at highest risk of postpartum loss to care, thereby reducing negative health consequences and HIV transmission. Our results clearly correlate certain individual-level factors (such as pre-pregnancy and postpartum obstetric engagement in care, or shorter time-period between delivery and first appointment) with improved postpartum RIC. We additionally show evidence that systemic and/or societal factors (such as health systems and societal changes in response to the global COVID pandemic) may more strongly impact RIC than other social factors traditionally viewed as important.

RIC is an important indicator that predicts adherence to ART and long-term health of PLWH. Pregnancy presents a unique opportunity to study RIC in PLWH, given the increased access to care and, often, increased motivation to stay engaged in care during pregnancy. The transition to lifelong HIV care after delivery can be challenging due to competing priorities. Previous studies of U.S. PLWH demonstrated low RIC rates in the first year postpartum (11–16). Late engagement in HIV care postpartum (12, 14) and poor antenatal HIV care (11) were associated with inadequate RIC, as also found in our study. On the other hand, previous research correlated depression, illicit drug use, and young maternal age with poor RIC, associations not found in our study to significantly contribute based on the Random Forest machine learning model despite 43% of our cohort reporting a mental health condition (83% of which was depression) and 17% reporting current substance use (Table 1) (11, 22).

We found a significant decline in RIC after 2020, which likely reflects the impact of the pandemic and/or the resultant changes to the health care system on RIC. This decline is also noted when compared to data from pre-pandemic data from a 2014 study of a similar regional population (16). The pandemic has had multiple well documented long-term effects on general health outcomes and health systems, and specifically for maternal and perinatal outcomes (23–25). One hypothesis regarding gaps in engagement in care after delivery include the loss of dedicated case management services that are often available during pregnancy. Losing case management at a time with so much change in insurance coverage and healthcare systems likely contributes to decreased RIC. The pandemic likely exacerbated these barriers. The pandemic's impact and health system's impact on postpartum RIC and other aspects of the HIV care continuum deserves dedicated further study.

Data regarding infant feeding choice was not included in the original data collection for this cohort. Regardless, as the Panel on Treatment of HIV in Pregnancy and Prevention of Perinatal Transmission in the U.S. improves support for patient-centered decision making regarding infant feeding, improving postpartum RIC will need to be emphasized and facilitated to reduce risk of lactational HIV transmission. Further study, especially via qualitative methodologies, is needed to evaluate the effect of infant feeding decision on RIC and maternal healthcare following delivery as this will become more significant going forward.

This study has several strengths, including that our large single-site but diverse cohort is well-characterized by detailed prenatal and postpartum demographic and clinical information. Furthermore, our contemporary study period captured a unique time span that included the COVID-19 pandemic and post-pandemic years, likely reflecting the pandemic's durable impact on the organization and utilization of the U.S. health care system. Additionally, our study has notable limitations. First, our study was retrospective and thus relied on the accuracy and scope of the electronic medical record (EMR) data accessible within our health care system, thereby excluding those pregnant PLWH who may have received care elsewhere. We attempted to minimize the impact of this by excluding anyone who planned to be seen outside our system after delivery. Second, while our cohort is diverse, our results may not be generalizable to other hospital systems outside of a large metropolitan area or without the capacity for multidisciplinary care within a large, academic hospital system. Finally, we limited our analyses to the first year postpartum and so were not able to estimate longer term effects of adequate care in the immediate postpartum period on overall morbidity.

Pregnancy is a unique life transition for PLWH, characterized by increased motivation and access to medical care and generally excellent engagement in HIV care. Most PLWH are able to achieve viral suppression by the time of delivery, benefiting both the pregnant person and the neonate. However, engagement in care dramatically decreases after pregnancy due to multiple complex barriers. Further qualitative efforts are needed to determine what exactly is contributing to improved engagement during pregnancy and what is changing after delivery. HIV care is life-long for all individuals; staying in care after delivery is not only essential in determining their own long-term health, but the health of their families and any future pregnancies. It is only through deeper understanding that effective interventions may be developed that could improve support for individuals during this difficult transition.

## Data Availability

The raw data supporting the conclusions of this article will be made available by the authors, without undue reservation.
